# Promoter DNA methylation contributes to human *β*-defensin-1 deficiency in atopic dermatitis

**DOI:** 10.1080/19768354.2018.1458652

**Published:** 2018-05-24

**Authors:** Yoo-Hun Noh, Jaehyouk Lee, Seong Joon Seo, Soon Chul Myung

**Affiliations:** a Agricultrual Research Center, Dankook University, Yongin, Republic of Korea; b Bio-Integration Research Center for Nutra-Pharmaceutical Epigenetics, Department of Urology, Chung-Ang University College of Medicine, Seoul, Republic of Korea; c Department of Dermatology, Chung-Ang University College of Medicine, Seoul, Republic of Korea

**Keywords:** Human *β*-defensin-1, DNA methylation, atopic dermatitis

## Abstract

Atopic dermatitis (AD) is a chronic inflammatory skin disease caused by epidermal barrier dysfunction and dysregulation of innate and adaptive immunity. Epigenetic regulation of human *β*-defensin-1 (HBD-1) might be associated with a variety of defects in the innate immune system during AD pathogenesis. We investigated the possible mechanism of decreased *HBD-1* gene expression in AD and demonstrated the restoration of HBD-1 transcription in undifferentiated normal human epidermal keratinocyte cells after treatment with a DNA methyltransferase inhibitor. We also conducted an *in vitro* methylated reporter assay using a reporter containing 14 CpG sites. Methylation of the 14 CpG sites within the HBD-1 5′ region resulted in an approximately 86% reduction in promoter activity and affected HBD-1 transcriptional regulation. We then compared methylation frequencies at CpG 3 and CpG 4 between non-lesional and lesional epidermis samples of patients with severe AD and between these paired tissues and healthy control epidermis from normal volunteers without AD history. Bisulfite pyrosequencing data showed significantly higher methylation frequencies at the CpG 3 and 4 sites in AD lesional samples than in non-lesional AD skin and normal skin samples (*P* < 0.05). These results suggest that the DNA methylation signature of HBD-1 is a novel diagnostic/prognostic marker and a promising therapeutic target for the compromised stratum corneum barrier attributed to HBD-1 deficiency.

## Introduction

Atopic dermatitis (AD) is a common chronic epidermal disorder characterized by an abnormal skin barrier. It is widely accepted that both the permeability barrier and the antimicrobial barrier are required to maintain the epidermal ensemble that protects against infectious skin diseases (Elias and Steinhoff 2008). While dysfunctions in the permeability barrier are mainly caused by the loss of filaggrin in the stratum corneum (SC), an aberrant antimicrobial barrier occurs in part due to a defect in the innate immune system, which includes antimicrobial peptides (AMPs) (McGirt and Beck 2006). AMPs play a key role in innate immune responses against diverse microbes (Selsted and Ouellette 2005). Human *β*-defensin-1 (HBD-1) is a unique AMP in its constitutive expression in various epithelial tissues, including the skin. Initial insights into antimicrobial barrier dysfunction in AD came from studies showing that reduction in AMPs might be associated with a variety of defects in the innate immune system during AD pathogenesis (Ong et al. 2002). HBD-2 and HBD-3 levels in lesional skin biopsy specimens from subjects with AD are lower than in those from psoriasis patients (Nomura et al. 2003). Additionally, *HBD-1* mRNA expression is considerably lower in the lesional skin of AD patients than in normal controls (Suga et al. 2014). Subjects with AD have increased susceptibility to cutaneous colonization and infection with various microorganisms, of which *Staphylococcus aureus* is the most common pathogen (McGirt and Beck 2006). Epigenetic mechanisms including DNA methylation have been implicated in a huge variety of human diseases correlated to gene−environment interactions. Reduction of AMPs via epigenetic alterations is one of the plausible explanations for *S. aureus* burden in AD patients (De Benedetto et al. 2009).

The aims of this study were first to provide further insight into the potential regulation mechanisms that could underlie the link between HBD-1 and AD. To evaluate whether epigenotyping for HBD-1 by the putative DNA methylation signature was applicable to skin specimens from AD subjects, we sought to characterize the DNA methylation patterns of HBD-1 in both atopic cases and controls.

## Materials and methods

### Cell culture and reagents

The normal human epidermal keratinocyte (NHEK) cell line was obtained from PromoCell (Heidelberg, Germany) and grown in Keratinocyte Growth Medium 2 (PromoCell) supplemented with Keratinocyte Growth Medium 2 SupplementMix (PromoCell). All cells were maintained at 37°C in a humidified incubator with 5% CO_2_. The undifferentiated NHEK cells were treated with 2′-deoxy-5-azacytidine (DAC) (Sigma-Aldrich, St. Louis, MO, USA) for 72 h.

### Tissue samples

Skin punch biopsy specimens were obtained from paired non-lesional and lesional epidermis of patients with severe AD (*n *= 10) (Table 2) and from healthy control epidermis of normal volunteers without a history of AD (*n *= 4). Control skin samples of healthy donors were obtained from the forearm lesions. Genomic DNA extraction was performed using the QIAamp DNA Mini Kit (Qiagen, Hilden, Germany). Each patient provided informed consent, and this study was approved by the institutional review board of the Chung-Ang University Hospital.

### Quantitative real-time PCR

Total RNA was isolated from cells using the RNeasy Mini Kit (Qiagen), following the manufacturer’s instructions, and cDNA synthesis for real-time two-step RT-PCR was performed with 1 ㎍ of total RNA using the QuantiTect Reverse Transcription Kit (Qiagen). Following this, 1 ㎍ of diluted cDNA was used for the cycling reactions using the Rotor-Gene SYBR Green PCR Kit (Qiagen), according to the manufacturer’s instructions. Amplification and quantitative analysis were performed in a Rotor-Gene Q 5plex HRM system (Qiagen).

### *In vitro* methylated reporter assay

**Figure 1.**
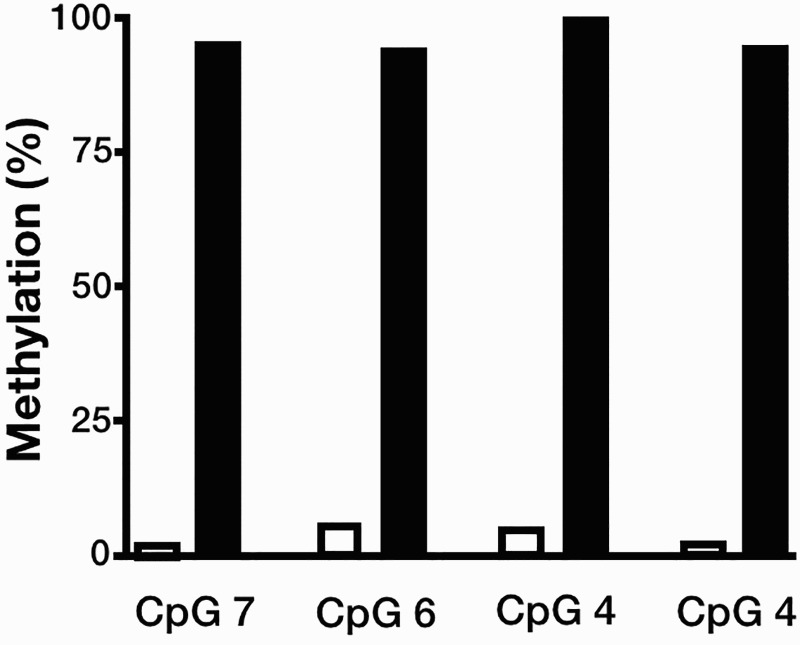
Quantitative methylation analysis of unmethylated and *in vitro* methylated luciferase reporter plasmids. Four CpG dinucleotides located in the *HBD-1* promoter were assessed by bisulfite pyrosequencing.

**Figure 2.**
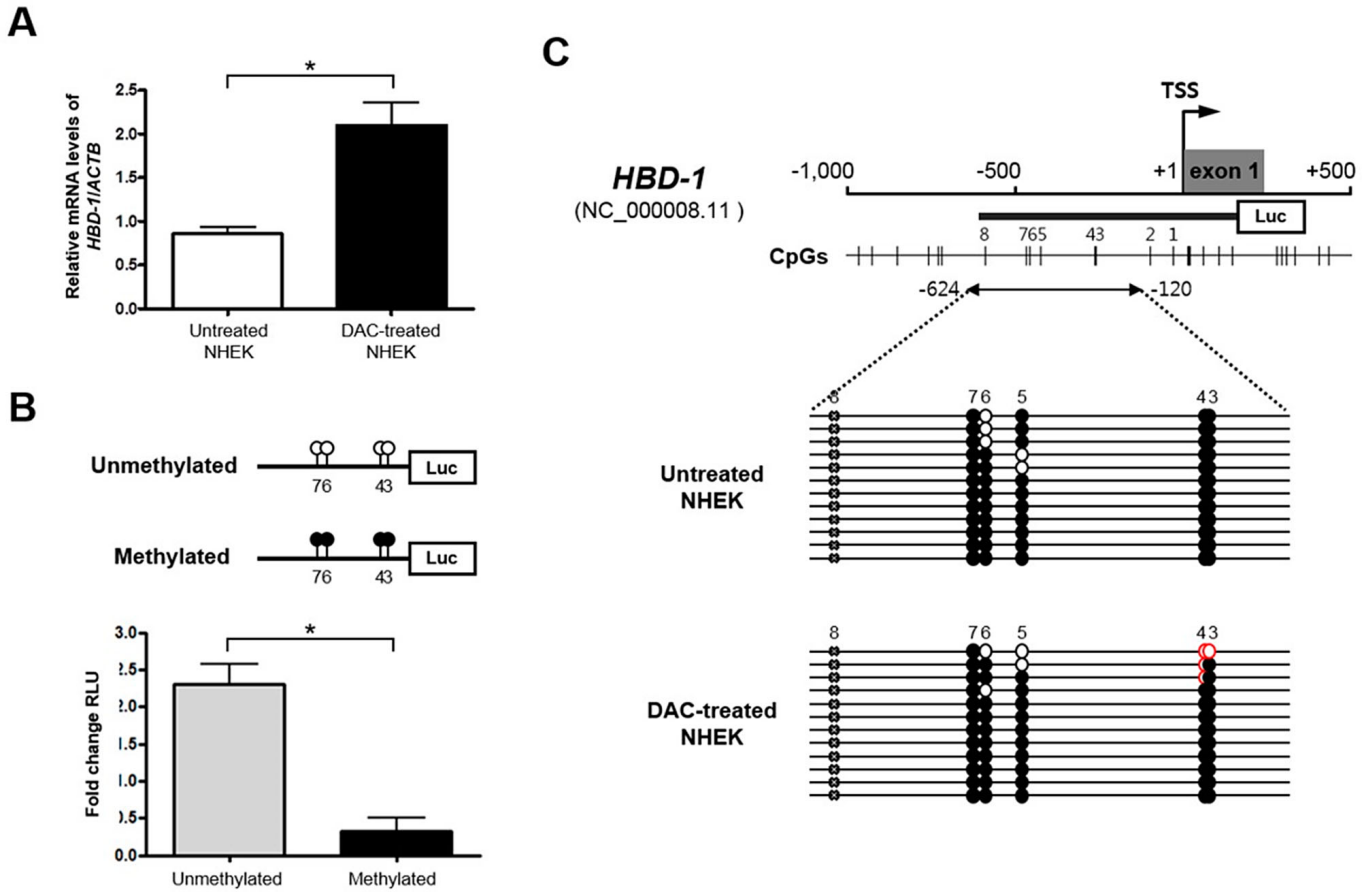
Epigenetic regulation of *HBD-1* in undifferentiated NHEK cells. **A**, Restoration of *HBD-1* mRNA by 2′-deoxy-5-azacytidine (DAC) in undifferentiated NHEK cells. **B**, Promoter reporter assay of the *HBD-1* 5′ region in HEK293 T cells using the pCpGfree-basic-Lucia reporter plasmid. **C**, Bisulfite sequencing analysis of the *HBD-1* 5′ region in undifferentiated NHEK cells. ○ Unmethylated and ● methylated CpG sites. *P < 0.05.

A sequence fragment of 790 bp, harboring 14 CpG sites located at the 5′-end region of the *HBD-1* gene (Figure 2, C), as well as a 5′ *Bam*HI site and a 3′ *Hin*dIII site, was synthesized and cloned into a pCpGfree-basic-Lucia (InvivoGen, San Diego, CA, USA) reporter plasmid that encodes a secreted coelenterazine-utilizing luciferase. Briefly, 5 ㎍ of the Lucia reporter vector was methylated *in vitro* with 12 U of M.*SssI* CpG methyltransferase (New England Biolabs, Ipswich, MA, USA) for 20 h at 37°C. After purification using the DNA Clean & Concentrator™-5 (Zymo Research, Irvine, CA, USA) according to the manufacturer’s protocol, plasmid methylation was verified by bisulfite pyrosequencing (Figure 1). Unmethylated or methylated reporter plasmids were transiently transfected in HEK293 T cells using jetPEI (Polyplus-transfection, New York, NY, USA). Luciferase activity of the Lucia reporter was measured from the supernatant of transfected cells at 72 h after transfection using the QUANTI-Luc assay reagent (InvivoGen). In each experiment, pCMV-CLuc 2 Control Plasmid (New England Biolabs), encoding the secreted *Cypridina* luciferase (CLuc), was co-transfected as a normalization control and luminescence was then determined from the supernatant using the BioLux *Cypridina* Luciferase Assay Kit (New England Biolabs). Reporter activity was normalized by calculating the ratio of Lucia to CLuc values.

### DNA methylation analyses

To perform bisulfite genomic sequencing analysis, genomic DNA was extracted from the NHEK cells using the QIAamp DNA Mini Kit (Qiagen), and the bisulfite modification of genomic DNA was performed using EZ DNA Methylation-Lightning™ Kit (Zymo Research), following the manufacturer’s instructions. The bisulfite-converted genomic DNA was then amplified using a primer set specific to the proximal promoter region of *HBD-1* (nucleotides from 624 to 120 bp upstream of the transcription start site (TSS) denoted as +1) containing six CpG sites. The cycling conditions were as follows: 10 min at 94°C; 45–50 cycles of 30 s at 94°C, 30 s at 52°C, and 30 s at 72°C; followed by 10 min at 72°C. PCR products were purified and subcloned into the pGEM-T Easy Vector (Promega) for subsequent sequencing reactions. Nucleotide sequences of 10–15 independent clones were analyzed.

The bisulfite-modified genomic DNA from patients with AD was amplified using primer sets specific to the *HBD-1* promoter region. Pyrosequencing reactions were performed using PyroMark Gold Q96 Reagents (Qiagen), and quantitative analysis was then performed on the PyroMark Q96 ID platform (Qiagen), following the supplier’s instructions. The primer sequences used in DNA methylation analyses are listed in Table 1.

**Table 1.** Primers used to amplify the *HBD-1* promoter region in PCR reactions for bisulfite genomic sequencing and bisulfite pyrosequencing analyses.

PCR reactions	5′ to 3′ sequences
Bisulfite genomic sequencing	
Forward primer	TTGGTAGGGTTGAAGTGGGAG
Reverse primer	TAAAACCCTAATACCAACTCCTC
Bisulfite pyrosequencing	
Forward primer	TTTTTGTAAGGGAAGAGGGTGAAG
Reverse primer	[Biotin]-TCACACTAAAATCCCTCCTTCTAAATCAC
Sequencing primer	AATTAAAGAGGTTAATATTAGTT

### Statistical analysis

To determine significant differences in the quantitative methylation data generated by pyrosequencing measurements, comparisons between non-lesional and lesional tissue samples were made using the Wilcoxon signed-rank test. Due to the abnormal distribution of methylation data, further analyses among skin tissue samples stratified by normal and AD were carried out using the Kruskal-Wallis test. A *P*-value <0.05 was considered statistically significant. Statistical analyses were conducted using IBM SPSS Statistics 20 (IBM Corp., Armonk, NY, USA).

## Results

To investigate whether epigenetic modulation of HBD-1 occurs in keratinocytes, which are one of the main sources of AMPs in human skin (Gallo and Nakatsuji 2011), we first examined HBD-1 transcription in undifferentiated NHEK cells after treatment with the DNA methyltransferase inhibitor DAC.

DNA demethylation by DAC induced a considerable increase in *HBD-1* mRNA expression in the NHEK cells (Figure 2, *A*). These data imply that *HBD-1* silencing in the undifferentiated NHEK cells is correlated with an epigenetic inactivation mechanism involving DNA methylation. To test whether the DNA methylation pattern in the 5′-end region of *HBD-1* is important for regulation of core promoter activity, we conducted an *in vitro* methylated reporter assay (Figure 2, *B*). According to a previous study showing the critical region for transcriptional activity of the *HBD-1* promoter (Sun et al. 2006), we synthesized the genomic sequence of *HBD-1* (NC_000008.11) (−612 to +165 bp relative to the TSS, +1) covering the region of the core promoter and exon 1 and containing 14 CpG sites (Figure 2, *C*). After cloning the *HBD-1* 5′ region into a CpG-free luciferase vector, *in vitro* methylation was performed to assess the role of CpG methylation in the regulation of *HBD-1* expression (Figure 2, *B*). The HEK293 T cells transfected with the methylated *HBD-1* reporter exhibited markedly lower luciferase activity than those transfected with the unmethylated *HBD-1* constructs. The approximately 86% reduction in promoter activity due to methylation of 14 CpG dinucleotides within the *HBD-1* 5′ region suggests that DNA methylation can affect *HBD-1* transcriptional regulation directly.

**Figure 3.**
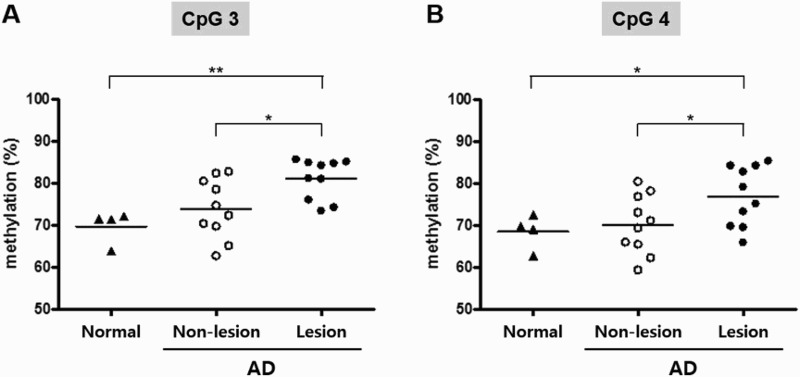
Quantitative methylation analysis of the **A**, CpG 3 and **B**, CpG 4 dinucleotide within the *HBD-1* promoter in normal individuals (*n* = 4) and patients with atopic dermatitis (AD) (*n* = 10). *P < 0.05 and **P < 0.01.

To evaluate whether epigenotyping for *HBD-1* by the putative DNA methylation signature within the *HBD-1* promoter is applicable to skin specimens from AD subjects, we compared methylation frequencies at both CpG 3 and CpG 4 between non-lesional and lesional epidermis samples of the same patients with severe AD (*n *= 10) (Table 2) and between these paired tissues and healthy control epidermis from normal volunteers without AD history (*n *= 4) (Figure 3). Bisulfite pyrosequencing data showed statistically significant differences in methylation frequencies between the non-lesional and lesional samples (*P *= 0.0156 for CpG 3 and *P *= 0.0493 for CpG 4). Furthermore, methylation frequencies at the CpG 3 and CpG 4 sites were significantly higher in lesional AD samples than in normal skin samples (*P *= 0.0047 for CpG 3 and *P *= 0.0475 for CpG 4) (Figure 3).

**Table 2.** Clinical characteristics of patients with atopic dermatitis.

Subjects	Sex/Age (yr)	EASI	IGE	Biopsy sites (NL/L)
1	M/22	19	2386	Back/Popliteal
2	M/47	17.8	NA	Forearm/Popliteal
3	M/42	25.2	NA	Thigh/Abdomen
4	F/19	20.5	206	Thigh/Neck
5	M/17	24.6	>5000	Abdomen/Antecubital
6	M/56	18.4	805	Back/Neck
7	M/32	16	>5000	Thigh/Neck
8	M/45	24	>5000	Back/Neck
9	M/31	17.9	1784	Back/Back
10	M/39	19.3	NA	Back/Flack

EASI, Eczema Area Severity Index; NL, non-lesion; L, lesion.

## Discussion

Epigenetic mechanisms involving DNA methylation are essential for normal development and function of the immune system. In recent studies, distinct tissue-specific patterns of DNA methylation associated with atopic dermatitis were screened in epidermal lesions. The results showed that CpGs assigned to *LRRC8C, S100A5*, and *EBP49* were hypermethylated in AD skin lesions (Rodriguez et al. 2014). These genes have been reported to have a partial association with epidermal differentiation and immune responses, but no association has been reported in the pathogenesis of AD. HBD-1 in human skin is a strong AMP, which may represent important cofactors in the pathogenesis of AD. Here, we showed that HBD-1 silencing in undifferentiated NHEK cells is regulated by an epigenetic inactivation mechanism involving DNA methylation of 14 CpG dinucleotides within the HBD-1 5′ region. In lesional AD skin, methylation frequencies at the CpG 3 and CpG 4 sites within the HBD-1 promoter were significantly higher than in normal skin.

To identify specific CpG sites crucial to *HBD-1* expression in the NHEK cells, we performed bisulfite genomic sequencing of a region upstream of the proximal promoter region of *HBD-1*. A previous report showed that methylation in both the proximal promoter (i.e. CpG 1 and CpG 2, assigning the first CpG dinucleotide 31 bp upstream of the *HBD-1* TSS as CpG 1) and exon 1 (containing six CpG sites) of the *HBD-1* 5′ region was not significantly different among the samples examined (Sun et al. 2006). Therefore, we determined the methylation profiles of the remaining six CpG dinucleotides (i.e. CpG 3 to CpG 8) that were initially involved in the *in vitro* methylated reporter assay. Since a single nucleotide polymorphism (rs2978863) is present in the CpG 8 locus (denoted by x) within the *HBD-1* promoter region (GenBank accession no. NC_000008.11) in the NHEK cell line, the other five CpG dinucleotides (CpGs 3 to 7) were subjected to bisulfite sequencing. As shown in Figure 2, C, the methylation profile of the *HBD-1* promoter revealed detectable demethylation at both CpG 3 and CpG 4 loci in the DAC-treated NHEK cells, compared with untreated control cells. Taken together, our results indicate that these differentially methylated single CpG units in the *HBD-1* promoter might be particularly important in transcriptional regulation of *HBD-1* in the NHEK cell line.

AD genotyping studies have mainly focused on the filaggrin gene. Here, we demonstrated that epigenetic modulation of the *HBD-1* promoter, i.e. DNA methylation in two single CpG units, could affect *HBD-1* expression *in vitro*. Our results also highlighted the lesional epidermis-specific hypermethylation of both CpG sites in paired skin biopsy samples of AD patients. Cutaneous innate immune defects resulting in increased *S. aureus* colonization in AD subjects might occur at least in part due to this epigenetic predisposition of constitutively expressed *HBD-1*. In conclusion, our findings suggest that the DNA methylation signature of *HBD-1* is a novel diagnostic/prognostic marker and a promising therapeutic target for the compromised SC barrier attributed to HBD-1 deficiency. Further research is necessary to determine the cause leading to the hypermethylation of *HBD-1* in patients’ skin with AD.
